# Expression Analysis of Hormone Receptor 38 (HR38) and Ecdysone-Induced Protein 75 (E75) Genes and Their Functional Implications in the Development of *Heortia vitessoides* Moore

**DOI:** 10.3390/biology15010044

**Published:** 2025-12-26

**Authors:** Na Liu, Hanyang Wang, Jiahe Liang, Zhiqiang Zhong, Tong Lin

**Affiliations:** College of Forestry and Landscape Architecture, South China Agricultural University, 483 Wushan Road, Guangzhou 510642, China; ln021045@stu.scau.edu.cn (N.L.); frisk990709@gmail.com (H.W.); hazelleung26@163.com (J.L.); zzq1255354017@126.com (Z.Z.)

**Keywords:** *Heortia vitessoides*, HR38, E75, molting and metamorphosis, 20-hydroxyecdysone (20E), pest management

## Abstract

HR38 and E75 are early 20-hydroxyecdysone (20E)-responsive nuclear receptors that play important roles in insect molting and metamorphosis. Here, we cloned and characterized HvHR38 and HvE75 from *Heortia vitessoides* and analyzed their conserved domains and phylogenetic positions. Both genes exhibited distinct stage- and tissue-specific expression patterns closely associated with ecdysteroid-regulated developmental processes. Hormone-induction assays further demonstrated that the transcription of Hv*HR38* and Hv*E75* was strongly activated by 20E. RNA interference targeting either gene resulted in significant transcript knockdown, accompanied by incomplete molting, pupal deformities, and molting failure, ultimately leading to markedly reduced survival, with dsHv*E75* causing the highest lethality. Collectively, these results suggest that HR38 and E75 function as key components of the early 20E-responsive transcriptional network involved in molting regulation, and highlight their potential as RNAi targets for species-specific and environmentally sustainable pest management.

## Simple Summary

Insect growth and development are controlled by steroid hormones that regulate molting and metamorphosis. Two hormone-responsive genes, HR38 and E75, act as key regulators in converting hormonal signals into developmental responses. However, their roles remain largely unexplored in many forest pests. In this study, we identified and characterized the HR38 and E75 genes in *Heortia vitessoides*, an important defoliating pest of agarwood-producing trees. Both genes showed clear stage- and tissue-specific expression patterns and responded rapidly to the molting hormone 20-hydroxyecdysone. When either gene was silenced using RNA interference, insects exhibited severe developmental abnormalities, including failed molting, malformed pupae, and reduced survival. These findings demonstrate that HR38 and E75 are essential for normal development in *Heortia vitessoides*. This work improves our understanding of hormone-regulated insect development and highlights HR38 and E75 as potential molecular targets for environmentally friendly pest control strategies.

## 1. Introduction

Insects rely on a highly coordinated endocrine system to regulate growth, molting, and metamorphosis. Among these hormonal signals, 20-hydroxyecdysone (20E) is the central steroid hormone that drives developmental transitions by activating the EcR/USP receptor complex and initiating a hierarchical cascade of early and late response genes [1,2,3,4]. This transcriptional cascade is further shaped by juvenile hormone (JH) and other physiological cues, ensuring that developmental timing is precisely synchronized with internal and environmental conditions [5,6,7]. Within this network, nuclear receptors such as Hormone receptor 38 (HR38) and Ecdysone-induced protein 75 (E75) function as key early 20E-responsive regulators that couple endocrine signals to downstream gene-expression programs essential for larval–pupal metamorphosis [4,8,9].

HR38 is the insect ortholog of the vertebrate NR4A nuclear receptor family and functions as an immediate early-response gene that is rapidly induced following 20E stimulation [8,10]. First identified in *Drosophila melanogaster*, HR38 plays crucial roles in neuronal remodeling, adult cuticle formation, behavioral plasticity, and the coordination of metamorphic transitions [10,11,12]. Functional studies across diverse insect lineages indicate that HR38 is a deeply conserved regulator of 20E signaling. In Lepidoptera, including *Bombyx mori*, *Heliothis virescens*, and *Spodoptera litura,* HR38 expression rises sharply during molting and drives epidermal reorganization and larval–pupal transition [13,14,15]. In Coleoptera, RNAi-mediated knockdown of HR38 in *Tribolium castaneum* disrupts apolysis and cuticle deposition, ultimately causing developmental arrest [16,17].

HR38 also mediates species-specific physiological processes. In insects, HR38 functions as an ecdysteroid-responsive transcription factor involved in peripheral and behavioral regulation. In Lepidoptera, such as *Spodoptera littoralis*, HR38 is rapidly induced by ecdysteroids in peripheral sensory tissues and contributes to the modulation of olfactory responsiveness, linking systemic 20E signaling to tissue-specific physiological and behavioral outputs [18]. In Hymenoptera, particularly in the honey bee *Apis mellifera*, HR38 expression is significantly upregulated during foraging activity, together with early growth response protein 1 and other downstream components of the ecdysteroid signaling pathway, suggesting a role for HR38 in coordinating endocrine signaling with behavior-associated physiological states [19]. In mosquitoes, including *Aedes aegypti* and *Anopheles gambiae*, HR38 participates in reproductive maturation, vitellogenic signaling, and post-blood-meal endocrine remodeling [20,21,22]. Additionally, HR38 functions as a conserved neuronal activity marker across insect taxa, linking endocrine signals to activity-dependent gene expression in the nervous system [23]. Collectively, these findings illustrate that HR38 acts as a multifunctional nuclear receptor that couples 20E signaling to developmental remodeling, physiological transitions, and reproductive processes across insects.

E75 is another primary 20E-inducible nuclear receptor that serves as a key decoder of ecdysone pulses and a coordinator of stage-specific gene expression programs [4]. Originally identified as the gene responsible for the 75B early puff in *Drosophila* polytene chromosomes, E75 is rapidly induced at the onset of each molting cycle [24]. Unlike most nuclear receptors, E75 contains a heme prosthetic group, enabling it to sense nitric oxide and oxygen levels and thereby integrate endocrine signals with cellular metabolic states [25].

Comparative studies demonstrate that the regulatory roles of E75 are conserved across major arthropod groups. In Lepidoptera, E75 isoforms (E75A/B/C) display distinct temporal patterns and contribute to larval–pupal transition, midgut remodeling, and autoregulatory control of ecdysteroid biosynthesis in *Bombyx mori* and *Galleria mellonella* [26,27,28,29]. In Diptera, particularly *Aedes aegypti*, E75 orchestrates vitellogenesis, ovarian maturation, and metabolic reprogramming after blood feeding [30,31,32]. In Coleoptera (*T. castaneum*), RNAi-mediated knockdown of E75 halts molting and leads to lethality [33]. Moreover, E75-like receptors in crustaceans such as *Metapanaeus ensis* also regulate molt initiation [34], underscoring an evolutionarily ancient role for E75 in arthropod endocrine control. Together, these findings position E75 as a heme-dependent integrator of hormonal and environmental signals, coordinating molting, metamorphosis, and reproduction across diverse arthropod lineages.

*Heortia vitessoides* (Lepidoptera: Crambidae) is a major defoliator of *Aquilaria sinensis*, an economically valuable tree species for agarwood production. Outbreaks of *H. vitessoides* cause severe defoliation, decrease tree vigor, and threaten the sustainability of agarwood resources. Repeated infestations not only impair plantation productivity but may also disrupt forest stand structure and plant–insect interactions. [35,36,37]. At present, management of *H. vitessoides* relies largely on chemical insecticides, which pose risks to non-target organisms and are inconsistent with the sustainable utilization of agarwood resources. Therefore, elucidating the molecular and endocrine mechanisms underlying its growth, development, and metamorphosis is of particular importance for advancing environmentally friendly pest control strategies.

HR38 and E75 are ecdysone-responsive nuclear receptors that play conserved roles in insect molting and metamorphosis. Functional studies in other lepidopteran pests, including *Helicoverpa armigera*, *Spodoptera litura*, and *Ostrinia furnacalis*, have demonstrated their involvement in hormone-mediated developmental regulation [19,22,33]. However, to date, HR38 and E75 have not been identified or functionally characterized in *H. vitessoides*. Accordingly, the present study represents the first molecular identification and functional analysis of HR38 and E75 in this species, providing new insights into its endocrine-regulated development and establishing a molecular foundation for RNAi-based, species-specific pest management.

RNA interference (RNAi) has emerged as a powerful tool for investigating insect gene function and offers promising avenues for species-specific pest management [38]. Functional studies in *T. castaneum*, *A. aegypti*, *S. exigua*, *B. mori*, and *N. lugens* demonstrate that nuclear receptor genes [16,17,31,32,39], including HR38, E75, EcR, and USP, are effective RNAi targets that disrupt molting, impair reproduction, and reduce survival [38,40]. Recent advances in dsRNA delivery, environmental risk assessment, and RNA-based biopesticides further support the feasibility of targeting developmental regulators in pest control frameworks [41,42,43,44,45].

In this study, the full-length cDNA sequences of Hv*HR38* and Hv*E75* were cloned and analyzed from *H. vitessoides*. Their molecular characteristics, evolutionary relationships, and temporal–spatial expression profiles were investigated, including transcriptional responses to 20E treatment. RNA interference assays were conducted to clarify their functional roles in larval molting and metamorphosis. This work broadens current understanding of the ecdysone-responsive transcriptional network in *H. vitessoides* and identifies HR38 and E75 as potential molecular targets for RNAi-based sustainable pest management.

## 2. Materials and Methods

### 2.1. Insects

*H. vitessoides* individuals were collected from Tianluhu Forest Park (Guangzhou, China) and subsequently reared under controlled laboratory conditions. The insects were maintained at 26 ± 1 °C, 75 ± 5% relative humidity, and a photoperiod of 14 h light and 10 h dark. Larvae were continuously fed with fresh leaves of *Aquilaria sinensis* (Lour.) Spreng. Eggs and larvae from the first to fifth instars were reared under these same environmental conditions. When *H. vitessoides* larvae reached the fifth (mature) instar, individuals were transferred into eight-compartment rearing boxes (each compartment measuring 6.2 cm × 4.7 cm × 4.5 cm). The bottom of each compartment was covered with a 2 cm layer of sterilized sand maintained at approximately 50% relative humidity to facilitate pupation. Larvae pupated and adults emerged within the sand layer. Newly emerged adults were transferred to mesh cages and provided with a 7% (*w*/*v*) honey solution as a food source.

### 2.2. Sample Preparation

For tissue-specific expression analysis, day 3 fifth-instar larvae (L5D3) of *H. vitessoides* with similar body size and developmental status were selected. After surface cleaning and sterilization, the larvae were dissected on ice under a stereomicroscope to obtain the following tissues: epidermis, midgut, fat body, hemolymph, and head. Adult samples were dissected using the same procedure, and the following tissues were collected: head, thorax, legs, wings, male abdomen, and female abdomen. To investigate the developmental expression profiles, individuals from different stages were collected, including larvae (L1–L5; L5D1–D4), prepupae (D1–D3), and adults (D1). All experiments included at least three biological replicates, and each replicate consisted of no fewer than 30 individuals. All collected samples were washed thoroughly with ice-cold phosphate-buffered saline (PBS, pH 7.4), immediately frozen in liquid nitrogen, and stored at −80 °C until total RNA extraction.

### 2.3. RNA Extraction and cDNA Synthesis

Total RNA was extracted from each sample using the Total RNA Kit II (Omega Bio-Tek, Norcross, GA, USA) following the manufacturer’s protocol. The concentration and purity of the extracted RNA were determined using a NanoPhotometer spectrophotometer (Implen, Munich, Germany). DNA was synthesized using the PrimeScript™ RT Reagent Kit with gDNA Eraser (Takara, Kusatsu, Shiga, Japan) according to the manufacturer’s instructions. The synthesized cDNA was stored at −20 °C until further use.

### 2.4. Sequence Verification and Phylogenetic Analysis

To identify the HR38 and E75 genes of *H. vitessoides*, the transcriptome database of *H. vitessoides* was searched using the keywords “HR38” and “E75”, respectively. All unigene clusters annotated as HR38 or E75 were retrieved, and the candidate sequences were subjected to nucleotide (BLASTn) and protein (BLASTx) searches on the NCBI platform (https://blast.ncbi.nlm.nih.gov/Blast.cgi (accessed on 21 May 2025)) to confirm sequence identity and obtain the most complete coding regions. The resulting sequences were designated as *HvHR38* (GenBank accession number: PV637195.1) and *HvE75* (GenBank accession number: PV637196.1).

The open reading frame (ORF) sequences of *HvHR38* and *HvE75* were determined using the ORF Finder tool (https://www.ncbi.nlm.nih.gov/orffinder/ (accessed on 27 May 2025)). Specific primers were designed based on the obtained cDNA sequences using Primer Premier 5.0 software (Premier Biosoft International, Palo Alto, CA, USA). PCR amplification was performed under the following conditions: initial denaturation at 98 °C for 3 min, followed by 15 cycles of 98 °C for 20 s, 66 °C for 10 s, and 72 °C for 15 s with a 1 °C decrease per cycle; then 25 cycles of 98 °C for 20 s, 52 °C for 10 s, and 72 °C for 15 s; followed by a final extension at 72 °C for 2 min and storage at 12 °C. PCR products were purified and sequenced to verify successful cloning of the target genes.

The physicochemical properties of *HvHR38* and *HvE75* proteins were predicted using the ExPASy ProtParam tool (http://web.expasy.org/protparam/ (accessed on 12 June 2025)), including theoretical isoelectric point (pI) and molecular weight (Mw). Potential N-glycosylation sites were analyzed with the NetNGlyc 1.0 Server (https://services.healthtech.dtu.dk/services/NetNGlyc-1.0 (accessed on 15 June 2025)), and protein secondary structures were predicted using JPred 4 (http://www.compbio.dundee.ac.uk/jpred/index.html (accessed on 17 June 2025)).

To investigate evolutionary relationships, amino acid sequences of *HR38* and *E75* homologs from other insect species were retrieved from NCBI, and two separate phylogenetic trees were constructed using the neighbor-joining (NJ) method implemented in MEGA 7.0 software (MEGA Limited, Auckland, New Zealand) with 1000 bootstrap replicates to assess branch reliability.

### 2.5. Primer Design and Quantitative Real-Time Polymerase Chain Reaction (RT-qPCR)

Specific primers were designed from the conserved regions of the target genes using Primer Premier 5.0 software (Premier Biosoft International, Palo Alto, CA, USA). Primer synthesis was outsourced to Guangzhou Tsingke Biotechnology Co., Ltd. (TsingkeBiotechnology Co., Ltd., Guangzhou, China). The primer sequences are listed in (Table 1). The previously synthesized cDNA was diluted appropriately within the range recommended by the manufacturer of the SYBR Green qPCR kit and used as a template for RT-qPCR. Quantitative PCR was carried out using a SYBR Green–based detection system with a 2× SYBR Green qPCR Premix (Universal) (Guangzhou XinKailai Biotechnology Co., Ltd., Guangzhou, China). in a final reaction volume of 20 μL, containing 10 μL of SYBR Green Premix, 0.4 μL of each forward and reverse primer (final concentration of 0.2 μM), 1–2 μL of cDNA template, and nuclease-free water to volume. Reactions were prepared under low-light conditions, sealed, and briefly centrifuged to remove air bubbles. RT-qPCR was performed on a LightCycler 480 II Real-Time PCR System (Roche, Basel, Switzerland). The thermal cycling conditions were as follows: initial denaturation at 95 °C for 30 s, followed by 40 cycles of 95 °C for 10 s and 60 °C for 30 s. Each reaction was carried out in triplicate (technical replicates), and *β*-actin was used as the internal reference gene for normalization.

### 2.6. dsRNA Preparation and Injection

Double-stranded RNA (dsRNA) was synthesized using the T7 RiboMAX™ Express RNAi System Kit (Promega, Madison, WI, USA) according to the manufacturer’s instructions. Specific primers containing the T7 promoter sequence were designed, and PCR amplification was performed to obtain the corresponding DNA templates. These templates were used to synthesize ds*HvHR38*, ds*HvE75*, and ds*GFP* fragments. Following synthesis, the DNA templates were removed, and the dsRNA products were annealed, while single-stranded RNA (ssRNA) was degraded. The final dsRNA was purified and dissolved in nuclease-free water. The concentration and purity of dsRNA were determined using a NanoPhotometer spectrophotometer (Implen, Munich, Germany). Each dsRNA was diluted to a final concentration of 5 µg/µL, and 1 µL was injected into the dorsal side of the penultimate abdominal segment of fourth-instar (L4) larvae using a microinjection syringe (Eppendorf, Hamburg, Germany). The control groups were injected with equivalent volumes of ds*GFP* or DEPC-treated water. Each treatment consisted of 30 larvae, with three independent biological replicates. Phenotypic changes and survival rates were monitored throughout the experimental period. The physiological condition of larvae was assessed daily by gently touching their bodies with a fine brush. Larvae that failed to respond within one minute were recorded as dead.

### 2.7. Juvenile Hormone III (JH III) and 20-Hydroxyecdysone (20E) Injection

To investigate the regulatory roles of HR38 and E75 in 20-hydroxyecdysone (20E) and juvenile hormone (JH) signaling pathways, hormone injection assays were conducted using different concentrations of 20E and JH III. Both hormones were initially dissolved in dimethyl sulfoxide (DMSO) to prepare concentrated stock solutions at a concentration of 10 mg/mL and stored at −20 °C until use. On the day of injection, the stock solutions were diluted with phosphate-buffered saline (PBS, pH 7.4) to obtain final concentrations of 0, 250, 500, 1000, and 2000 ng/μL. The final concentration of DMSO was kept constant among all treatments (DMSO: PBS = 1:99, *v*/*v*). The 0 ng/μL group served as a solvent control and contained PBS with the same DMSO concentration but without hormone. All solutions were mixed thoroughly and kept on ice before use. A 1 μL volume of each hormone solution was injected into the dorsal side of fourth-instar (L4) larvae of *H. vitessoides* using a microinjection syringe (Eppendorf, Hamburg, Germany). After injection, larvae were maintained under standard rearing conditions, and samples were collected at 3, 6, 12, and 24 h post-injection. The collected samples were immediately frozen in liquid nitrogen and stored at −80 °C for subsequent quantitative real-time PCR (RT-qPCR) analysis to assess the relative expression levels of *HvHR38* and *HvE75* in response to hormone treatment.

### 2.8. Statistical Analysis

The experimental data were initially processed using Microsoft Excel software (Microsoft Corp., Redmond, WA, USA). The relative expression levels of target genes were calculated using the 2^−ΔΔCt^ method [46]. One-way analysis of variance (ANOVA) was performed using SPSS 18.0 software (IBM Corp., Armonk, NY, USA) to evaluate differences among developmental stages and tissues, followed by Tukey’s multiple comparison test. Statistical significance was set at *p* < 0.05. All results are presented as the mean ± standard error (SE).

## 3. Results

### 3.1. Sequence Analysis of HvHR38, HvE75, and Phylogenetic Analysis

The gene sequence was searched in the transcriptome of *H. vitessoides*. After BLAST homology alignment via the NCBI website (https://blast.ncbi.nlm.nih.gov/Blast.cgi (accessed on 21 May 2025)), the complete sequence of the HR38 and E75 gene was obtained and named HvHR38 (Gen Bank accession number: PV637195.1), HvE75 (Gen Bank accession number: PV637196.1). The full-length sequence of the HR38 gene consists of 4664 bp, with an open reading frame (ORF) of 1834 bp encoding 610 amino acids. With the help of the online program ProtParam, the predicted molecular weight of the HR38 protein is 67.31 kDa, with a theoretical isoelectric point (pI) of 7.21. The protein contains 61 acidic amino acids (Asp and Glu) and 61 basic amino acids (Arg and Lys). The full-length sequence of the E75 gene is 2801 bp, with an ORF of 2268 bp encoding 756 amino acids. The predicted molecular weight of the E75 protein is 83.42 kDa, with a theoretical pI of 9.04. The protein comprises 85 acidic amino acids (Asp and Glu) and 102 basic amino acids (Arg and Lys).

**Figure 1:**
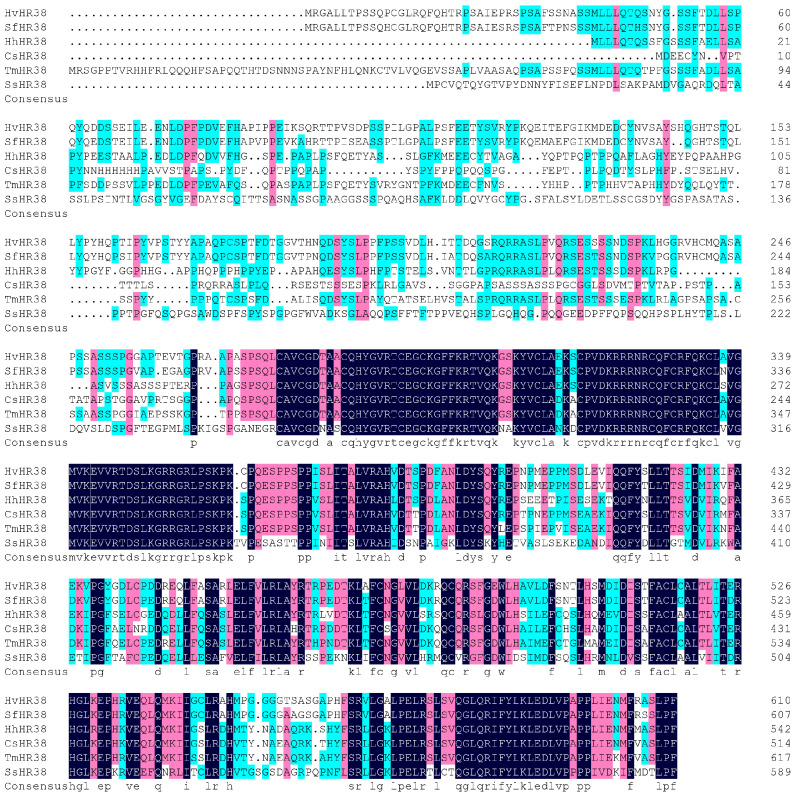
Sequence alignment of Hv*HR38* with insect homologs. The amino acid residues that are identical in all sequences are marked with dark shading, whereas light shading indicates that at least 75% amino acids are identical in all sequences. The aligned sequences are the predicted amino acid sequences of HR38 from *H. vitessoides* (Hv*HR38* PV637195.1), *Spodoptera frugiperda* (Sf*HR38* XP_035446230.1), *Halyomorpha halys* (Hh*HR38* XP_014290140.1), *Cryptotermes secundus* (Cs*HR38* XP_033606170.1), *Tenebrio molitor* (Tm*HR38* XP_068903538.1), *Salmo salar* (Ss*HR38* XP_014022904.1).

**Figure 2:**
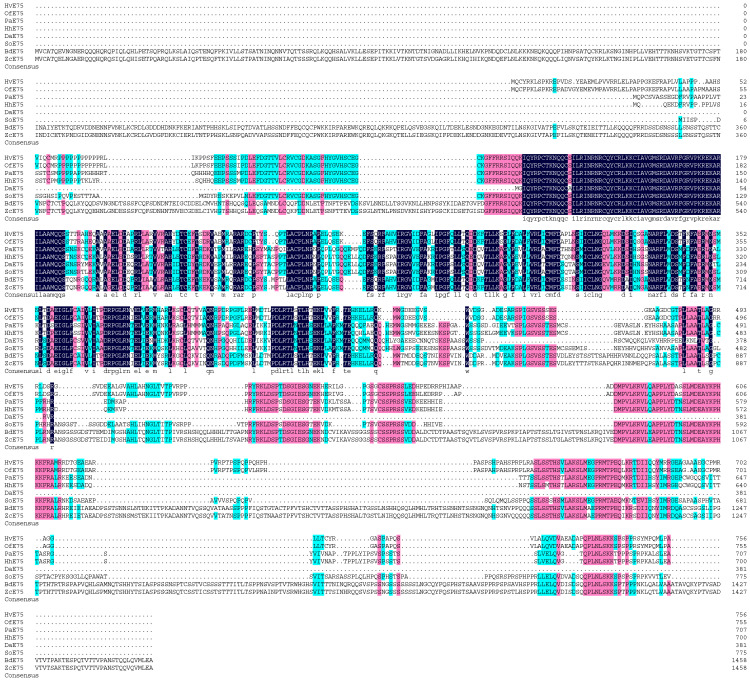
Sequence alignment of Hv*E75* with insect homologs. The amino acid residues that are identical in all sequences are marked with dark shading, whereas light shading indicates that at least 75% amino acids are identical in all sequences. The aligned sequences are the predicted amino acid sequences of E75 from *H. vitessoides* (Hv*E75* PV637196.1), *Ostrinia furnacalis* (Of*E75* XP_028166113.1), *Pyrrhocoris apterus* (Pa*E75* WIM36146.1), *Halyomorpha halys* (Hh*E75* XP_014276624.1), *Dendroctonus armandi* (Da*E75* URZ62289.1), *Sitophilus oryzae* (So*E75* XP_030746896.1), *Bactrocera dorsalis* (Bd*E75* XP_049315306.1), *Zeugodacus cucurbitae* (Zc*E75* XP_028895193.1).

Multiple sequence alignment revealed that the HR38 and E75 proteins of *Heortia vitessoides* share a high degree of evolutionary conservation with their homologs in other species. The HR38 protein of *H. vitessoides* showed strong homology with those of *Spodoptera frugiperda* (92.13%), *Halyomorpha halys* (66.38%), *Cryptotermes secundus* (62.67%), *Tenebrio molitor* (67.05%), and *Salmo salar* (60.66%) (Figure 1). Similarly, the E75 protein exhibited high sequence conservation with homologs from *Ostrinia furnacalis* (96.18%), *Pyrrhocoris apterus* (55.15%), *Halyomorpha halys* (62.01%), *Oncopeltus fasciatus* (56.56%), *Bactrocera dorsalis* (62.17%), *Zeugodacus cucurbitae* (56.58%), and *Rhagoletis zephyria* (66.99%) (Figure 2). These results indicate that both HR38 and E75 are highly conserved nuclear receptor proteins, suggesting that their regulatory functions in hormonal signaling and metamorphosis are evolutionarily maintained across diverse insect taxa.

**Figure 3:**
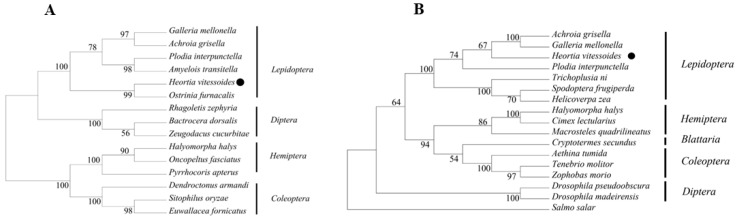
(**A**) Phylogenetic analysis of Hv*HR38.* The predicted amino acid sequences of Hv*HR38* to gether with 17 selected HR38 members were aligned, and a phylogenetic tree was constructed using MEGAX. The HR38 of *Heortia vitessoides* is marked with black circles. GenBank accession numbers are as follows: *Achroia grisella* (XP_059060612.1); *Galleria mellonella* (XP_026757160.1); *Heortia vitessoides* (PV637195.1); *Plodia interpunctella* (XP_053625936.1); *Trichoplusia ni* (XP_026727300.1); *Spodoptera frugiperda* (XP_035446230.1); *Helicoverpa zea* (XP_047042384.1); *Halyomorpha halys* (XP_014290140.1); *Cimex lectularius* (XP_014249767.1); *Macrosteles quadrilineatus* (XP_054283667.1); *Cryptotermes Secundus* (XP_033606170.1); *Aethina tumida* (XP_019872449.1); *Tenebrio molitor* (XP_068903538.1); *Zophobas morio* (XP_063903367.1); *Drosophila pseudoobscura* (XP_001356865.4); *Drosophila madeirensis* (BFF95704.1); *Salmo salar* (XP_014022904.1). (**B**) Phylogenetic analysis of Hv*E75*. The predicted amino acid sequences of Hv*E75* to gether with 15 selected E75 members were aligned, and a phylogenetic tree was constructed using MEGAX. The E75 of *Heortia vitessoides* is marked with black circles. GenBank accession numbers are as follows: *Galleria mellonella* (XP_026758166.1); *Achroia grisella* (XP_059054396.1); *Plodia interpunctella* (XP_053625982.1); *Amyelois transitella* (XP_013195908.1); *Heortia vitessoides* (PV637196.1); *Ostrinia furnacalis* (XP_028166113.1); *Rhagoletis zephyria* (XP_017468203.1); *Bactrocera dorsalis* (XP_049315306.1); *Zeugodacus cucurbitae* (XP_028895193.1); *Halyomorpha halys* (XP_014276624.1); *Oncopeltus fasciatus* (ABP02024.1); *Pyrrhocoris apterus* (WIM36146.1); *Dendroctonus armandi* (URZ62289.1); *Sitophilus oryzae* (XP_030746896.1); *Euwallacea fornicates* (XP_066157284.1).

Phylogenetic trees were constructed based on the amino acid sequences of HR38 and E75 from *H. vitessoides* and representative insect species across Lepidoptera, Hemiptera, Coleoptera, and Diptera (Figure 3A,B). In the HR38 phylogenetic tree, *H. vitessoides* clustered closely with other lepidopteran species, including *Plodia interpunctella*, *Spodoptera frugiperda*, and *Helicoverpa zea*, forming a well-supported lepidopteran clade. This clade was clearly separated from those comprising hemipteran, coleopteran, and dipteran species, indicating a high degree of evolutionary conservation of HR38 at the order level within Lepidoptera. Within this clade, the overall topology showed short genetic distances among lepidopteran HR38 sequences, further supporting the conserved nature of this nuclear receptor across lepidopteran insects. These results suggest that HR38 maintains a stable evolutionary position within Lepidoptera, consistent with its conserved role in ecdysteroid-responsive developmental processes.

Similarly, phylogenetic analysis of E75 showed that *H. vitessoides* clustered with lepidopteran species such as *Galleria mellonella* and *Ostrinia furnacalis* with high bootstrap support, forming a well-supported lepidopteran clade. This clade was clearly separated from dipteran species, including *Bactrocera dorsalis* and *Rhagoletis zephyria*, indicating that E75 exhibits a conserved phylogenetic distribution at the order level. This clustering pattern is consistent with sequence homology analysis and supports the conclusion that E75 is a highly conserved nuclear receptor among insects.

Taken together, the phylogenetic analyses of HR38 and E75 demonstrate that both genes occupy stable and conserved evolutionary positions within Lepidoptera, consistent with their conserved roles as ecdysone-responsive nuclear receptors.

### 3.2. Stage-Specific and Tissue-Specific Expression Patterns of HvHR38, HvE75

**Figure 4:**
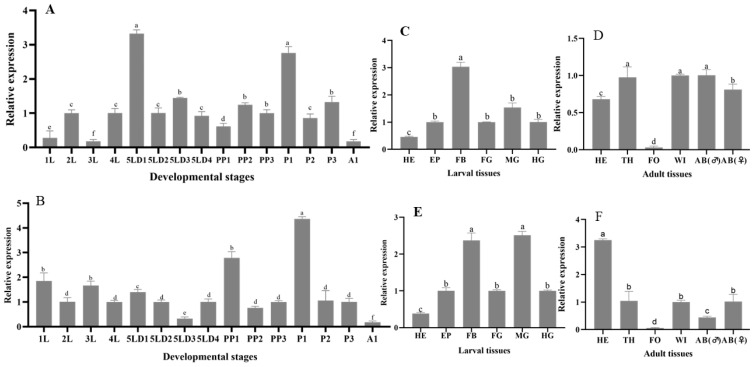
(**A**,**B**) Developmental expression patterns of Hv*HR38* (**A**) and Hv*E75* (**B**) at different stages: L1–L4 (first to fourth instar larvae), L5D1–L5D4 (fifth instar larvae at days 1 to 4), PP (prepupa), P1–P3 (pupa at days 1 to 3), A1 (1-day-old adult). Expression levels are shown relative to first-instar larvae (L1). (**C**,**E**) Tissue-specific expression of *HvHR38* (**C**) and *HvE75* (**E**) in larval tissues (fifth instar larvae), Expression levels are shown relative to the larval head tissue. including HE (head), EP (epidermis), FB (fat body), FG (foregut), MG (midgut), and HG (hindgut). Expression levels are shown relative to the larval head tissue. (**D**,**F**) Tissue-specific expression of *HvHR38* (**D**) and *HvE75* (**F**) in adult tissues, including HE (head), TH (thorax), FO (foot), WL (wing), AB (♂) (male abdomen), and AB (♀) (female abdomen). Expression levels are shown relative to the adult head tissue. Error bars represent the mean ± standard error (SE) of three biological replicates. Different letters above the bars indicate significant differences (*p* < 0.05), determined by one-way ANOVA followed by Tukey’s test.

We used the RT-qPCR method to investigate the relative expression pattern of Hv*HR38* and Hv*E75* in different developmental stages and tissues of *H. vitessoides*. Hv*HR38* exhibited distinct stage-specific expression patterns during the development of *H. vitessoides*. Its expression peaked at the fourth-instar larvae (4L) and rose again at the prepupal stage (PP3), while reaching the lowest levels at the second-instar (2L) and adult stage (A1) (Figure 4A), showing a clear bimodal trend. This suggests that HR38 may function as an early-response gene in the ecdysone signaling cascade. In tissue-specific expression, Hv*HR38* was highly expressed in the FB (fat body) of larvae, followed by the MG (midgut) and HE (head) (Figure 4C), implying its potential involvement in energy metabolism and developmental regulation. In adults, Hv*HR38* was predominantly expressed in the HE and WI (wing) (Figure 4D), suggesting possible roles in hormonal response and adult behavioral regulation.

Hv*E75* displayed a clear temporal expression pattern, with its highest level at the prepupal stage (PP3) and significantly reduced expression at the fifth-instar day 3 (5LD3) and adult stage (A1), indicating that Hv*E75* is strongly induced during the larval–pupal transition (Figure 4B). In tissue distribution, Hv*E75* was highly expressed in the FB and MG of larvae, while showing low levels in the EP (epidermis) and HG (Figure 4E). In adults, Hv*E75* showed its highest expression in the HE and AB (female abdomen), but low expression in the TH (thorax) and FO (foot) (Figure 4F). These results suggest that Hv*E75* participates in the 20E signaling pathway, playing essential roles in energy metabolism, developmental regulation, and reproduction.

In summary, both Hv*HR38* and Hv*E75* exhibited distinct temporal and spatial expression patterns during the development of *H. vitessoides*. Their expression levels increased significantly before pupation, indicating that both genes are involved in the 20E-mediated developmental transition. Hv*HR38* functions as an early-response factor activated during the initiation of molting signals, while Hv*E75* acts as a downstream effector strongly induced in later stages. Their high expression in the FB fat body and MG midgut suggests crucial roles in metabolic regulation and energy supply, jointly modulating molting and metamorphosis in *H. vitessoides*.

### 3.3. Hormone-Induced Expression Responses of HvHR38 and HvE75 to 20E and JH Injection

**Figure 5:**
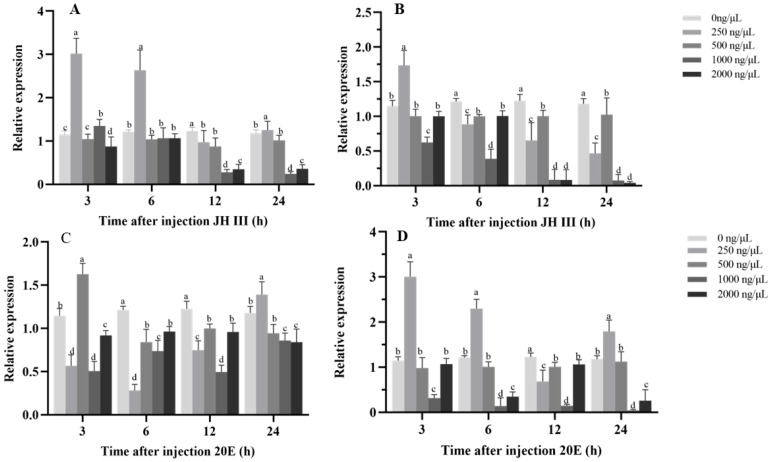
Effects of hormone injections on gene expression in larvae. (**A**,**B**) Relative expression of Hv*HR38* and Hv*E75* after injection of JH III at various concentrations (0, 250, 500, 1000, 2000 ng/µL) and time points (3 h, 6 h, 12 h, 24 h). (**C**,**D**) Relative expression of Hv*HR38* and Hv*E75* after injection of 20E at various concentrations (0, 250, 500, 1000, 2000 ng/µL) and time points (3 h, 6 h, 12 h, 24 h). Different letters above error bars indicate significant differences (*p* < 0.05). Expression levels are shown relative to the 0 ng/μL treatment group. Error bars represent the mean ± standard error (SE) of three biological replicates.

The hormone-induction assays revealed distinct yet interconnected expression response patterns of Hv*HR38* and Hv*E75* following juvenile hormone III (JH III) and 20-hydroxyecdysone (20E) treatments, reflecting their differential sensitivity to endocrine cues in *H. vitessoides*. Under JH III treatment, both genes exhibited a biphasic expression pattern characterized by early induction at low doses and marked suppression at higher doses and later time points. This response was particularly evident for Hv*HR38*, which showed strong upregulation within 3 h at 250–500 ng/μL, but pronounced inhibition at 1000–2000 ng/μL (Figure 5A). This pattern suggests that JH III may transiently stimulate the expression of certain 20E-responsive genes while suppressing their expression under elevated hormone levels or prolonged exposure. Hv*E75* displayed a similar but more sensitive expression profile, with stronger suppression observed at mid-to-late time points, which is consistent with the well-documented antagonistic effect of JH on early ecdysone-responsive gene expression during molting transitions (Figure 5B).

In contrast, 20E treatment resulted in rapid and robust induction of both genes, with expression peaks occurring within 3–6 h at moderate concentrations. The induction of Hv*E75* was stronger and more sustained, consistent with its classification as a canonical early 20E-responsive gene directly activated by ecdysone receptor signaling (Figure 5C). HvHR38 also showed a pronounced response to 20E but returned more rapidly toward basal expression levels, suggesting a transient role in early transcriptional responses rather than sustained activation (Figure 5D).

Taken together, these results indicate that Hv*HR38* and Hv*E75* exhibit distinct hormone-responsive expression dynamics under 20E and JH III treatments. Their differential induction and suppression patterns suggest that these two genes participate in coordinating the temporal regulation of endocrine-responsive gene expression during developmental transitions in *H. vitessoides*.

### 3.4. Silencing of HvHR38, HvE75 via RNAi

**Figure 6:**
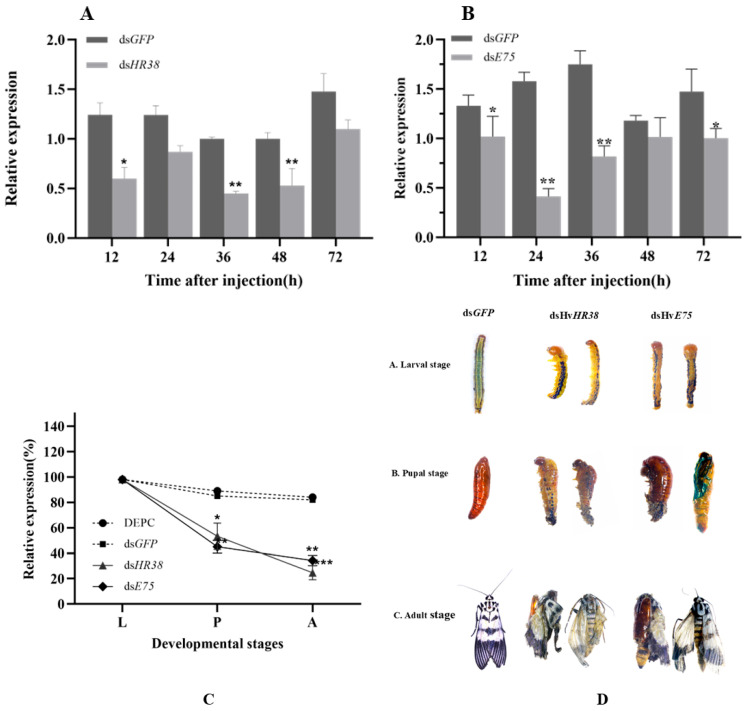
Effects of dsRNA injection targeting *HR38* and *E75* on the expression, survival, and lethal phenotypes of larvae, pupae, and adults. (**A**,**B**) Relative expression levels of HR38 (**A**) and E75 (**B**) in larvae at 12, 24, 36, 48 and 72 h post-injection with dsRNA targeting HR38 (dsHv*HR38*) and E75 (dsHv*E75*). Expression levels are shown relative to dsGFP-injected at the corresponding time points. (**C**) Survival rates of larvae (L), pupae (P), and adults (A) after injection with different treatments: DEPC, ds*GFP*, dsHv*HR38*, and dsHv*E75*. (**D**) Representative images of larvae, pupae, and adults after injection with ds*GFP* (control), dsHv*HR38*, and dsHv*E75*, showing developmental stages (L: larvae, P: pupae, A: adults) and the corresponding lethal phenotypes. Data are expressed as mean ± standard error (SE) of three biological replicates. Statistical significance was determined by one-way ANOVA followed by Tukey’s test. Significant differences are indicated by: * *p* < 0.05*,* ** *p* < 0.01, *** *p* < 0.001.

dsRNA was injected into L4D1 (first day of four instar) larvae, and total RNA was then extracted. The expression level of Hv*HR38* and Hv*E75* after RNAi was determined via RT-qPCR. The results showed that ds*HR38* and ds*E75* could silence the target gene (Figure 6A,B). Compared with dsGFP-treated, RNAi targeting Hv*HR38* and Hv*E75* produced a clear and stage-specific pattern of transcriptional suppression. For Hv*HR38*, transcript levels showed an immediate decline within 12 h post-injection and reached maximal suppression between 36–48 h, indicating that RNAi becomes fully effective during mid-to-late post-injection intervals. In contrast, Hv*E75* exhibited an even stronger and more persistent knockdown response. Expression decreased markedly as early as 12 h, dropped to its lowest level at 24 h, and remained significantly reduced through 48–72 h. This deeper and prolonged suppression of E75 suggests that it may be more sensitive to dsRNA-mediated degradation or possesses a faster mRNA turnover rate than HR38. The temporal profiles of both genes demonstrate that RNAi efficiency is not uniform across time but instead peaks during the 24–48 h window, with E75 showing consistently stronger responsiveness. These results confirm the reliability of the RNAi system in *H. vitessoides* and provide a precise timeline for evaluating downstream transcriptional or physiological effects following dsRNA treatment.

### 3.5. Phenotypic Analysis and Survival Assay After RNAi

Silencing Hv*HR38* and Hv*E75* produced a coordinated pattern of transcriptional suppression, developmental defects, and reduced survival, demonstrating their essential regulatory roles in the ecdysone-mediated metamorphic cascade. Both genes maintained near-normal expression during the larval stage, but their transcript levels declined sharply in the pupal and adult stages, indicating that RNAi becomes most effective during hormonally driven developmental transitions. This stage-specific knockdown coincided with progressive morphological abnormalities, including cuticle shrinkage and incomplete molting in larvae, malformed and collapsed pupae, and frequent eclosion failures in adults (Figure 6C,D). The severity of these defects paralleled the strength of gene suppression, particularly in the dsHv*E75* group, which exhibited the lowest transcript abundance and the most dramatic morphological disruption. Correspondingly, survival rates decreased continuously from larva to adult, with only 20~30% of individuals completing metamorphosis. Collectively, these results show that HR38 and E75 act as indispensable early 20E-responsive transcription factors, whose reduced expression disrupts endocrine signaling and results in cumulative failure of molting, pupation, and eclosion. This integrated phenotype expression response underscores the pivotal contribution of HR38 and E75 to the developmental robustness of *H. vitessoides* and highlights their potential as sensitive molecular targets for RNAi-based pest management.

Collectively, the RNAi phenotypes indicate that Hv*HR38* and Hv*E75* are indispensable for normal metamorphosis and cuticle formation in *H. vitessoides,* likely functioning as key regulators within the 20-hydroxyecdysone (20E) signaling pathway.

## 4. Discussion

### 4.1. Molecular Characteristics and Evolutionary Conservation of HvHR38 and HvE75

The molecular characterization and phylogenetic analysis revealed that both Hv*HR38* and Hv*E75* encode nuclear receptor proteins with conserved C4-type zinc finger domains typical of ecdysone pathway members. HR38 lacks a classical ligand-binding domain, consistent with reports describing it as an orphan nuclear receptor [7,8]. E75 contains a heme-binding ligand-binding domain, enabling rapid 20E responsiveness [4,6]. Phylogenetic clustering with Lepidoptera orthologs such as *Ostrini furnacalis* and *Bombyx mori* further supports strong evolutionary conservation. These findings are consistent with previous studies showing that HR38 and E75 exhibit high conservation in insects, serving as essential components in steroid hormone-mediated transcriptional cascades [1,2,3,4].

### 4.2. Developmental Expression Patterns and Physiological Implications

Both Hv*HR38* and Hv*E75* showed stage-specific expression, peaking during pupation and adult emergence—periods requiring intensive ecdysteroid-driven remodeling. Similar expression patterns have been documented in *Spodoptera litura* and *Bombyx mori* [15,27]. Their enrichment in epidermis and midgut tissues suggests roles in cuticle synthesis, digestive remodeling, and metabolic coordination during metamorphosis. This pattern suggests that both genes are involved in 20E-dependent developmental reprogramming [10,11,14]. Moreover, tissue-specific expression analysis revealed abundant transcripts in the epidermis and midgut, which are primary targets of ecdysone signaling, implying a role in cuticle formation, digestion, and energy metabolism during developmental transitions [13,29].

### 4.3. Hormonal Regulation and Transcriptional Dynamics Following RNAi

HR38 knockdown resulted in early suppression followed by compensatory rebound, a feedback pattern consistent with previous observations in *Drosophila* [10]. E75 responded strongly to 20E induction, confirming its primary-response function [26]. Phenotypic defects after dsRNA treatment, molting failure, malformed pupae, and eclosion arrest, align with known functions of HR38 and E75 in coordinating 20E-dependent transcription [20,21,22,30,31,32,33]. The sustained reduction in survival further indicates that *H. vitessoides* is highly sensitive to perturbations in these regulators, and their functional deficiency can lead to cumulative developmental impairments throughout metamorphosis. Collectively, these findings confirm that the proper expression of HR38 and E75 is indispensable for successful larval-pupal-adult transition, and highlight their potential as promising RNAi-based targets for environmentally friendly pest management [39,41].

### 4.4. Comparative Functional Analysis of HR38 and E75 Across Insect Taxa

RNAi-based functional studies of HR38 and E75 homologs across insect taxa provide compelling evidence for the functional conservation of these nuclear receptors. In lepidopteran insects, silencing of E75 homologs has been shown to disrupt larval–pupal transitions, impair molting processes, and increase mortality, indicating a conserved role in mediating ecdysone-responsive developmental events [27,28,32]. Similarly, RNAi suppression of HR38 or HR38-like nuclear receptors in Lepidoptera has been associated with altered growth, delayed development, and dysregulated hormone-responsive gene expression [13,15], highlighting their involvement in endocrine-controlled developmental pathways.

Evidence from non-lepidopteran insects further reinforces this conserved functional pattern. In dipteran species such as *Drosophila melanogaster*, E75 functions as a canonical early-response gene within the ecdysone signaling cascade, coordinating developmental timing, metabolic homeostasis, and redox regulation [24], while HR38 is implicated in endocrine signaling and xenobiotic-responsive transcriptional regulation [11,12]. RNAi-mediated knockdown of HR38- or E75-related genes in coleopteran insects has likewise resulted in developmental retardation, abnormal molting, and reduced survival [16,33]. Collectively, these RNAi-based functional analyses across diverse insect orders substantiate the evolutionary conservation of HR38 and E75 mediated regulatory functions.

In *H. vitessoides*, the observed expression dynamics and RNAi phenotypes of HR38 and E75 are consistent with these conserved roles, suggesting that these nuclear receptors participate in a broadly shared hormonal regulatory framework. Their coordinated action with core components of the ecdysone signaling pathway, including EcR and USP, likely represents a conserved molecular mechanism underlying insect development across taxa [5].

### 4.5. Future Perspectives and Implications for Green Pest Management

The demonstrated functional conservation of HR38 and E75 across insect taxa highlights their potential as robust molecular targets for next-generation pest management strategies. RNAi-based approaches targeting these nuclear receptors offer a high degree of species specificity and reduced environmental risk compared with conventional chemical insecticides [40]. In *H. vitessoides*, the essential roles of HR38 and E75 in hormone-mediated development make them particularly attractive candidates for RNAi-based population control.

Future pest management strategies may integrate bacterially expressed dsRNA or nanoparticle-based delivery systems to enhance RNAi stability, uptake efficiency, and field applicability [41,43]. Moreover, combining RNAi-based gene silencing with biological control agents, such as entomopathogenic fungi (*Beauveria bassiana* and *Metarhizium anisopliae*), could generate synergistic effects, leading to enhanced insect mortality while preserving ecological balance. Such integrated approaches align well with the principles of green pest management by minimizing chemical inputs and non-target effects.

## 5. Conclusions

In this study, we comprehensively characterized the nuclear receptor genes *HvHR38* and *HvE75* in *H. vitessoides* and demonstrated their essential roles in ecdysone-mediated developmental regulation. Both genes exhibited distinct stage- and tissue-specific expression patterns and responded rapidly to 20E stimulation, confirming their identities as early ecdysone-responsive transcription factors [1,2,3,4]. JH III exerted an opposite and dose-dependent regulatory influence, further illustrating the antagonistic interplay between JH and 20E in coordinating developmental transitions [5,6,8]. RNA interference targeting either gene resulted in significant transcriptional suppression accompanied by progressive developmental defects, including incomplete molting, pupal malformation, and molting failure, ultimately leading to markedly reduced survival. The stronger knockdown response and more severe phenotypes observed in *HvE75* silenced insects highlight its particularly critical role within the early endocrine regulatory cascade.

Overall, this work deepens the understanding of nuclear receptor function in Lepidopteran insects and provides a molecular basis for RNAi-based pest management. Targeting HR38 and E75 offers promising potential for environmentally compatible control strategies. Future research integrating advanced RNAi delivery systems (such as nanoparticles or plant-mediated gene silencing) may advance sustainable, species-specific, and ecologically safe approaches for forest pest control [41,42,43].

## Figures and Tables

**Table 1:** Primers used for RT-qPCR and synthesis of ds*HR38*, ds*E75* and ds*GFP*.

Primer Name	Forward(5′-3′)	Reverse (5′-3′)	TM	Product Length (bp)
Hv*HR38*	CCTGGCTACGGAGATTTATG	GTATCTTCTGGGCGAGTGC	54.95/56.26	107
Hv*E75*	GACCGACAGGTCTCCTAA	GTTCGTAAATCGGGAAGGTAT	57.37/55.27	146
T7+dsHv*HR38*	Taatacgactcactataggg ^1^ CCACCAACCTACAATACATAC	taatacgactcactataggg CGCAAGTTCGTACTCCGTAAG	68.69/69.90	407
T7+dsHv*E75*	taatacgactcactataggg TTCCTACGGAGCTGATGGA	taatacgactcactataggg CGGAACCGGGGCCTAATATC	69.16/68.57	445
T7+ds*GFP*	taatacgactcactataggg CAGTTCTTGTTGAATAGATG	taatacgactcactataggg TTTGGTTTGTCTCCCATGATG	71.50/75.50	400
*β*-actin	GTGTTCCCCTCTATCGTGG	TGTCGTCCCAGTTGGTGAT	57.32/55.11	119

^1^ Lowercase letters indicate the T7 promoter sequence.

## Data Availability

The nucleotide sequences generated in this study are available in the NCBI database. The other original contributions presented in this study are included in the article. Further inquiries may be directed to the corresponding author.

## Abbreviations

The following abbreviations are used in this manuscript:

HR38	Hormone Receptor 38
E75	Ecdysone-Induced Protein 75
20E	20-hydroxyecdysone
JH	juvenile hormone
